# Effects of covariates and interactions on a genome-wide association analysis of rheumatoid arthritis

**DOI:** 10.1186/1753-6561-3-s7-s84

**Published:** 2009-12-15

**Authors:** Rector Arya, Elizabeth Hare, Inmaculada del Rincon, Christopher P Jenkinson, Ravindranath Duggirala, Laura Almasy, Agustin Escalante

**Affiliations:** 1 Division of Clinical Epidemiology, Department of Medicine, University of Texas Health Science Center, 7703 Floyd Curl Drive, San Antonio, Texas 78229, USA; 2 Medical Center Research Division, Edinburg-Regional Academic Health Center, University of Texas Health Science Division, 1214 West Schunior, Edinburg, Texas 78541, USA; 3 Department of Psychiatry, University of Texas Health Science Center, 7703 Floyd Curl Drive, San Antonio, Texas 78229, USA; 4 Division of Clinical Immunology, Department of Medicine, University of Texas Health Science Center, 7703 Floyd Curl Drive, San Antonio, Texas 78229, USA; 5 Division of Diabetes, Department of Medicine, University of Texas Health Science Center, 7703 Floyd Curl Drive, San Antonio, Texas 78229, USA; 6 Department of Genetics, Southwest Foundation for Biomedical Research, 7620 NW Loop 410, San Antonio, Texas 78245, USA

## Abstract

While genetic and environmental factors and their interactions influence susceptibility to rheumatoid arthritis (RA), causative genetic variants have not been identified. The purpose of the present study was to assess the effects of covariates and genotype × sex interactions on the genome-wide association analysis (GWAA) of RA using Genetic Analysis Workshop 16 Problem 1 data and a logistic regression approach as implemented in PLINK. After accounting for the effects of population stratification, effects of covariates and genotype × sex interactions on the GWAA of RA were assessed by conducting association and interaction analyses. We found significant allelic associations, covariate, and genotype × sex interaction effects on RA. Several top single-nucleotide polymorphisms (SNPs) (~22 SNPs) showed significant associations with strong *p*-values (*p *< 1 × 10^-4 ^- *p *< 1 × 10^-24^). Only three SNPs on chromosomes 4, 13, and 20 were significant after Bonferroni correction, and none of these three SNPs showed significant genotype × sex interactions. Of the 30 top SNPs with significant (*p *< 1 × 10^-4 ^- *p *< 1 × 10^-6^) interactions, ~23 SNPs showed additive interactions and ~5 SNPs showed only dominance interactions. Those SNPs showing significant associations in the regular logistic regression failed to show significant interactions. In contrast, the SNPs that showed significant interactions failed to show significant associations in models that did not incorporate interactions. It is important to consider interactions of genotype × sex in addition to associations in a GWAA of RA. Furthermore, the association between SNPs and RA susceptibility varies significantly between men and women.

## Background

Rheumatoid arthritis (RA) is a chronic inflammatory disease of the joints, of unknown cause. It affects around 2% of people 60 years and older, but occurs in people of all ages. Women are more susceptible to RA than are men, and the disease is more frequent in some Native North American groups [1]. Inflammation in RA centers on the joints, causing joint swelling, pain, and degradation of joint cartilage and bone.

Genetic factors influence RA susceptibility [2,3]. Although family and twin studies suggest a genetic contribution to RA susceptibility of about 50-60%, causative genetic variants have not been identified [4,5]. Furthermore, genome-wide linkage studies of both discrete and continuous traits (RF-IgM and anti-CCP phenotypes) have shown strong evidence for linkages with several loci including chromosomes 6 and 18. Moreover, some genetic systems are strongly linked to disease susceptibility and to the disease phenotype. Recent association studies have implicated the HLA region on 6p, which accounts for about 30% of heritable risk [6]. The most studied gene associated with joint damage in RA is *HLA-DRB1 *[7]. However, genes from non-HLA regions are largely unknown.

Given that genome-wide association analysis (GWAA) is explicitly designed to detect genetic variants under the common-disease common-variant (CDCV) model for complex traits such as RA, it was expected that GWAA would capture most common genetic variation in RA. Because RA results from the interplay between an individual's genetic background and unknown environmental factors, it is important to assess the effects of environmental factors and their interactions on RA. Using Genetic Analysis Workshop (GAW) 16 data, we assessed the effects of covariates and interactions on the GWAA of RA. The only such factor available in the dataset we studied was sex. Although sex is not itself an environmental factor, it can serve as a model for how environmental variables and gene × environment interactions could be treated in the GWAA of RA. In addition to being a strong RA susceptibility factor, there is published evidence of heterogeneity by sex in the association of certain genetic systems.

## Methods

### Subjects

For this GAW16 analysis, the GAW16 RA data (Problem 1) from the North American Rheumatoid Arthritis Consortium (NARAC) cases (*n *= 868) and matched controls (*n *= 1194) have been used. For the NARAC study, patients were drawn from rheumatology clinics across North America who were anti-CCP-positive and met the criteria for RA adopted by the American College of Rheumatology in 1987. The NARAC samples (*n *= 2062) were from multiplex families in which at least one sibling had obvious erosions as seen on radiography of the hand, and at least one sibling had an onset of RA between the ages of 18 and 60 years.

### Genotyping

As described in Padyukov et al., [8] SNP genotyping was performed at the Feinsterin Institute for Medical Research Samples according to the Illumina Infinium 2 assay manual (Illumina, San Diego). All cases and most of the control samples were genotyped with the HumanHap550k beadchip.

### Association analysis

We performed a population-based genome-wide association analysis using PLINK, a tool set for whole genome association [9]. We analyzed GAW16 Problem 1 data using a variety of analyses: stratification, association, and interaction analyses using PLINK software [9]. We used the structured association approach, a simple but powerful approach, to detect population stratification, as implemented in the PLINK software [9-11]. PLINK's clustering approach is based on the genome-wide average proportion of alleles shared identical-by-state (IBS) between two individuals, i.e., pairing up individuals based on genetic identity. IBS clustering is used to test whether two individuals belong to the same population. Following the stratification analysis, we performed a standard case-control association test using a Cochran-Mantel-Haenszel statistic (1 df) that tests for single-nucleotide polymorphism (SNP)-disease association conditional on the clustering i.e., accounts for stratification effects. We used the most stringent Bonferroni correction (BONF) as well as the less stringent Benjamini and Hochberg false discovery rate (BH-FDR) for multiple testing corrections.

### Interaction analysis

The covariates for a discrete trait (RA affection status) included sex and genotypic models: additive (ADD), dominant deviation (DD), and general (GM). An additive model represents the additive effects of SNPs i.e., the effect of each additional minor allele as represented by the direction of the regression coefficient. For example, a positive regression coefficient indicates that the minor allele increases risk. A DD model represents a separate test of the dominance component, and a general model represents the joint test of both ADD and DD components. However, in contrast to a dominance model, DD refers to a variable coded in such a way (0, 1, 0 for three genotypes AA, Aa, aa) that it represents the dominance deviation from additivity without specifying whether a particular allele is dominant or recessive. Effects of genotype × sex interactions: ADD × Sex and DD × Sex were assessed using a logistic regression approach as implemented in PLINK.

## Results

**Figure 1:**
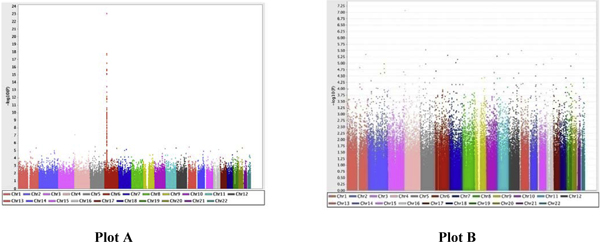
**Association plot of RA NARAC SNP data with HLA (Plot A) and without HLA (Plot B) regions**.

Using RA case status as affected and unaffected, we performed a general association and genotype × sex interaction analyses to identify those loci associated with RA. Given that the NARAC sample was stratified due to the presence of subpopulations, we performed the association analyses accounting for the population stratification. We found significant allelic associations, covariate (sex), and genotype × sex interaction effects on RA. The results of the association analyses are presented in Figure 1 and Table 1. In Figure 1, plot A shows associations across the entire genome and plot B shows associations in non-HLA regions. In Table 1, top SNPs (~22 SNPs with the best SNP on each chromosome) with strongest *p*-values (ranging from *p *< 1 × 10^-4 ^to *p *< 1 × 10^-24^) were presented along with corrected *p*-values for multiple testing, and genotype × sex interaction *p*-values. As shown in Table 1, the most strongly associated SNP (*p *< 1 × 10^-8^) in a non-HLA region was on chromosome 4, rs512244 (*p *= 8.362 × 10^-8^). Only three SNPs on chromosomes 4, 13, and 20 survived the overly conservative Bonferroni correction, and none of these three SNPs showed significant genotype × sex interactions. Interestingly, the Benjamini and Hochberg's false discovery rate (BHFDR), an alternative multiple testing correction, also yielded very similar *p*-values as shown in Tables 1 and 2.

**Table 1:** Genome-wide association of SNPs with RA in NARAC sample, best SNP on each chromosome

Chr	SNP	Position	Minor allele/major allele	Association *p*-value	Allelic OR (95% CI)	Bonferroni adjusted *p*-value	BH-false discovery rate *p*-value	Interaction (ADD × sex) *p*-value
1	rs865682	223,080,777	A/G	4.545 × 10^-6^	0.39(0.26-0.60)	0.16	0.16	-
2	rs12477038	201,518,282	G/A	1.038 × 10^-5^	0.47(0.33-0.66)	0.41	0.25	0.09
3	rs6775137	193,649,851	A/C	2.124 × 10^-5^	0.65(0.53-0.79)	0.70	0.70	-
4	rs512244	12,775,151	G/A	8.362 × 10^-8^	2.2(1.7-3.0)	0.002	0.002	0.14
5	rs7726943	67,854,337	G/A	2.991 × 10^-6^	0.62(0.50-0.76)	0.09	0.09	0.21
6	rs660895	32,685,358	G/A	8.346 × 10^-24^	3.4(2.7-4.3)	2.642 × 10^-19^	2.642 × 10^-19^	5.263 × 10^-45^
7	rs17470799	100,768,534	A/G	6.937 × 10^-6^	0.4(0.27-0.60)	0.18	0.12	1.0
8	rs966561	6,642,801	A/G	6.817 × 10^-5^	0.68(0.56-0.82)	1.0	0.54	0.93
9	rs306772	121,171,909	A/G	3.559 × 10^-5^	1.7(1.3-2.1)	0.83	0.40	0.09
10	rs7072006	133,595,618	A/G	5.309 × 10^-6^	0.48(0.35-0.67)	0.13	0.13	0.78
11	rs761453	31,834,576	A/G	3.867 × 10^-5^	0.61(0.48-0.77)	0.91	0.46	0.52
12	rs2283275	2,054,821	G/A	4.362 × 10^-6^	0.59(0.47-0.74)	0.10	0.10	0.98
13	rs17086849	28,064,399	A/G	3.158 × 10^-6^	0.56(0.44-0.72)	0.06	0.06	0.94
14	rs12885166	92,195,035	A/C	1.139 × 10^-5^	1.6(1.3-1.9)	0.18	0.18	0.45
15	rs11857639	71,424,825	A/G	1.083 × 10^-5^	0.39(0.25-0.61)	0.16	0.16	1.0
16	rs1076251	73,326,313	A/C	6.524 × 10^-6^	0.59(0.46-0.74)	0.10	0.10	0.21
17	rs1039519	3,394,664	G/A	7.11 × 10^-5^	1.52(1.2-1.9)	0.90	0.60	-
18	rs2174899	72,420,808	G/A	3.671 × 10^-5^	0.57(0.44-0.75)	0.54	0.54	-
19	rs9630874	57,619,851	A/C	1.27 × 10^-5^	0.31(0.17-0.54)	0.10	0.10	0.51
20	rs1182531	57,826,397	A/C	4.314^-6^	0.56(0.43-0.71)	0.05	0.05	0.17
21	rs12626622	31,981,460	A/G	2.063 × 10^-4^	1.8(1.3-2.4)	1.0	0.52	0.20
22	rs3830104	35,033,124	G/A	3.974 × 10^-5^	2.3(1.5-3.5)	0.29	0.21	0.73

**Table 2:** Covariate and genotype × sex interaction effects on RA in NARAC sample

			*p*-Value								
			
Chr	SNP	Position	Unadj	Bonferroni	BH-FDR	Sex	ADD	DD	General model	ADD × Sex	DD × Sex
1	rs9729157	19,363,907	0.88	1.0	0.99	0.6	0.09	0.91	0.23	3.029 × 10^-4^	0.02
1	rs11211044	44,987,845	0.03	1.0	0.82	4.614 × 10^-4^	0.17	0.02	0.004	0.86	7.327 × 10^-5^
2	rs280734	35,016,040	0.96	1.0	0.99	0.02	0.03	0.20	0.06	6.828 × 10^-5^	0.04
2	rs4953717	43,186,551	0.96	1.0	0.99	0.65	0.47	0.01	0.02	0.02	8.957 × 10^-6^
3	rs1391769	69,656,092	0.96	1.0	0.98	0.01	0.02	0.31	0.06	7.111 × 10^-5^	0.04
3	rs17032482	1,045,039	0.46	1.0	0.97	0.61	0.05	1.752 × 10^-4^	4.623 × 10^-4^	0.15	1.239 × 10^-4^
4	rs6811287	9,857,092	0.13	1.0	0.95	0.003	0.02	0.63	0.04	6.938 × 10^-6^	0.10
4	rs12646171	24,755,192	0.38	1.0	0.97	0.38	0.99	0.12	0.25	0.26	7.627 × 10^-5^
5	rs7725288	36,364,063	0.85	1.0	0.99	0.003	0.02	0.54	0.04	2.19 × 10^-6^	0.688
5	rs6595726	126,044,143	0.44	1.0	0.97	0.049	0.13	0.02	0.04	0.2027	1.234 × 10^-4^
6	rs493871	33,673,452	0.60	1.0	0.98	0.008	0.02	0.92	0.08	1.989 × 10^-5^	0.64
6	rs1023107	14,138,449	0.74	1.0	0.98	0.54	0.16	0.019	0.06	6.545 × 10^-4^	3.56 × 10^-4^
7	rs12718890	54,528,684	0.96	1.0	0.98	0.009	0.07	0.13	0.11	1.146 × 10^-4^	0.12
8	rs10090327	87,115,021	0.75	1.0	0.99	0.02	0.05	0.35	0.15	5.127 × 10^-5^	0.02
8	rs4873802	144,691,998	0.11	1.0	0.98	0.62	0.02	1.956 × 10^-6^	8.096 × 10^-6^	0.008	0.07
9	rs1413334	80,148,584	0.43	1.0	0.96	0.57	0.004	0.005	0.007	1.319 × 10^-4^	0.03
9	rs10738881	32,153,679	0.09	1.0	0.88	0.67	9.614 × 10^-5^	0.02	4.968 × 10^-4^	0.002	5.619 × 10^-5^
10	rs12412942	2,347,702	0.05	1.0	0.84	1.277 × 10^-4^	3.007 × 10^-4^	0.9816	8.523 × 10^-4^	3.414 × 10^-4^	0.42
11	rs1528648	14,096,262	0.66	1.0	0.99	0.006	0.08	0.13	0.16	1.219 × 10^-4^	0.17
12	rs6539583	75,591,349	0.36	1.0	0.95	0.24	0.18	0.34	0.36	7.358 × 10^-5^	0.07
13	rs1773126	46,123,327	0.26	1.0	0.96	0.02	0.10	0.74	0.21	1.307 × 10^-4^	0.80
14	rs4294750	104,034,158	0.35	1.0	0.95	0.30	6.582 × 10^-4^	0.10	0.002	8.144 × 10^-5^	0.06
15	rs2472297	72,814,933	0.20	1.0	0.92	0.87	4.194 × 10^-6^	0.04	1.389 × 10^-5^	2.078 × 10^-4^	0.006
16	rs12934235	5,637,396	0.62	1.0	0.98	0.02	0.33	0.64	0.23	8.075 × 10^-4^	0.11
17	rs225218	27,923,447	0.92	1.0	0.99	0.003	0.17	0.33	0.21	9.103 × 10^-6^	0.42
18	rs906283	10,918,707	0.15	1.0	0.99	0.008	9.993 × 10^-4^	0.24	0.002	9.886 × 10^-5^	0.36
19	rs12151188	44,147,374	0.40	1.0	0.96	0.75	0.01	0.78	0.03	7.579 × 10^-4^	0.03
20	rs6030315	35,003,238	0.96	1.0	0.99	0.20	0.03	0.76	0.11	6.594 × 10^-5^	0.15
21	rs991985	38,128,024	0.66	1.0	0.99	0.002	0.10	0.33	0.05	1.996 × 10^-4^	0.59
22	rs2880494	26,206,836	0.92	1.0	0.99	0.605	0.11	0.58	0.28	2.832 × 10^-4^	0.02

^a^Chr, chromosome; Unadj, Unadjusted *p*-values for main-effects association

In the genotype × sex interaction analysis, we identified a new set of SNPs to be highly significant in the presence of genotype × sex interaction, which showed no significance in the allelic association model. In Table 2, top SNPs showing significant interaction effects were presented along with corresponding association *p*-values for comparison. Out of 30 top SNPs with significant interactions (*p *< 1 × 10^-4 ^to *p *< 1 × 10^-6^) shown in Table 2, ~23 SNPs showed ADD × Sex and ~5 SNPs showed only DD × Sex interactions. Interestingly, the evidence of significance was reduced for most of the top SNPs showing association with RA in Table 1 in the presence of interactions as shown in Table 2. On the other hand, most of the SNPs showing highly significant effects of genotype × sex interactions (Table 2) showed no significant associations with RA (Table 1). Among covariates, two SNPs (on chromosomes 1 and 10) showed highly significant sex effects (*p *< 1 × 10^-4^), five SNPs showed additive effects, and two SNPs showed DD effects (Table 2). The observed differences between men and women in RA susceptibility may be attributable to the differences according to sex in their susceptibility to the disease and in the expression of clinical phenotype of RA.

## Discussion

In this study, we performed a population-based GWAA and genotype × sex interaction analyses using the GAW16 RA data (Problem 1) from NARAC. We performed association analyses without correcting for population stratification and found interesting associations (results not shown). Because the NARAC populations consisted of substructures, we repeated association analyses correcting for stratification, which made a significant difference in the association results. For example, in the first association analysis, a SNP (rs2476601) in the *PTPN22 *gene, an excellent candidate gene on chromosome 1 for RA, showed a highly significant (1.784 × 10^-12^) association with RA but the signal disappeared in the subsequent analysis after correction for stratification effects (*p *= 4.748 × 10^-4^). Furthermore, majority of the SNPs turned out to be insignificant after correcting for multiple testing using the Bonferroni correction, an overly conservative approach, and the BH-FDR, a less stringent correction that tolerates more false positives.

Following the main-effects association analysis, we performed interaction analyses to assess the effects of covariates and genotype × sex interactions on GWAA of RA. Sex was the only "environmental" covariate available to us. Although sex does not reflect environmental exposure in the traditional sense of a factor external to the individual, sex does significantly influence a person's internal environment in terms of hormonal actions and the like. Sex may also influence a person's exposure to external substances such as hair dyes, cosmetics, and fragrances. We found significant covariate and interaction effects on RA. Interestingly, SNPs showing significant main-effects associations did not show significant interactions. In contrast, the SNPs that showed the strongest evidence for interactions did not show significant main-effect associations. This finding reveals that in a GWAA, it is important to consider genotype (additive or dominant) by sex interaction effects on RA in addition to main-effects associations. Otherwise, such variants may be missed. In other words, the list of SNPs that would be followed up for replication or confirmation changes with the genotype × sex interaction effects.

On the other hand, SNPs with significant genotype × sex interaction did not necessarily have a significant (or even suggestive) main effect association. Therefore, limiting interaction tests to markers with significant main effects would likely find different results. We cannot say whether this would lose power or would protect against false positives on the basis of these results because the underlying genetic architecture of RA is largely unknown. For example, outside of the HLA region, there are 69 *p*-values of 10^-5 ^or better for the standard association analysis, so the top 100 SNPs would certainly be different if chosen using both standard association and genotype × sex interaction. However, genotype × sex interaction increases the multiple testing problem, and an exceedingly low *p*-value may be required to be considered significant after correction. In this study, the association between SNPs and RA susceptibility varies significantly between men and women. These results further support our earlier observation that there was significant heterogeneity between men and women, in the susceptibility and severity effects of HLA-DRB1, men being more susceptible to this gene system's influence [12].

## Conclusion

This case-control GWAA has yielded genomic regions exhibiting significantly different genotype frequencies between cases and controls that may contain genetic variants that predispose to RA and the regions identified may differ in the analyses with and without interactions. Our findings suggest that the association between SNPs and RA susceptibility varies significantly between men and women. In this GWAA of RA, characterization of how genes and the environment interact is important because the effects of covariates and genotype × sex interactions on RA are significant. Similar tests for interaction with sex and other environmental variables should be included in future case-control design GWAA in RA. Our study also emphasizes the importance of accounting for population stratification in analyzing GWAS data. Simple IBS-based cluster analysis changed the outcome of the analysis substantially for some markers by accounting for the effects of stratification due to the presence of subpopulations.

## List of abbreviations used

ADD: Additive; BH-FDR: Benjamini and Hochberg false discovery rate; BONF: Bonferroni correction; CDCV: Common-disease common-variant; DD: Dominant deviation; GAW: Genetic Analysis Workshop; GM: General; GWAA: Genome-wide association analysis; IBS: Identical-by-state; NARAC: North American Rheumatoid Arthritis Consortium; RA: Rheumatoid arthritis; SNP: Single-nucleotide polymorphism.

## Competing interests

The authors declare that they have no competing interests.

## Authors' contributions

RA conceived of the study, participated in its design, performed the statistical analyses including genome-wide association and interaction analysis and interpretation of results, and drafted the manuscript. EH participated in the statistical analysis. IDR participated in study design, funding, and editing manuscript. CPJ and RD helped in candidate gene identification and assisted in the data analysis. LA participated in the study design, helped in the genome-wide association analysis and interpretation of the results, and helped to draft the manuscript. AE participated in the design of the study, funding, assistance with data analysis, and editing manuscript. All authors read and approved the final manuscript.
